# Inflammatory profiles in Chilean Mapuche and non-Mapuche women with gallstones at risk of developing gallbladder cancer

**DOI:** 10.1038/s41598-021-83300-2

**Published:** 2021-02-11

**Authors:** Sarah S. Jackson, Vanessa Van De Wyngard, Ruth M. Pfeiffer, Paz Cook, Allan Hildesheim, Ligia A. Pinto, Sharon H. Jackson, Kelvin Choi, Ricardo A. Verdugo, Mara Cuevas, Cristian Yáñez, Eduardo Tobar-Calfucoy, Rocío Retamales-Ortega, Juan Carlos Araya, Catterina Ferreccio, Jill Koshiol

**Affiliations:** 1 Infections and Immunoepidemiology Branch, Division of Cancer Epidemiology and Genetics, National Institutes of Health, National Cancer Institute, Rockville, MD USA; 2 Facultad de Medicina, Pontificia Universidad Católica de Chile, Santiago, Chile; 3 Advanced Center for Chronic Diseases (ACCDiS), FONDAP, Santiago, Chile; 4 Frederick National Laboratory for Cancer Research, National Cancer Institute, Frederick, MD USA; 5 Division of Intramural Research, National Institute on Minority Health and Health Disparities, Bethesda, MD USA; 6 Programa de Genética Human, ICBM, Facultad de Medicina, Universidad de Chile, Santiago, Chile; 7 Departamento de Oncología Básico Clínica, Facultad de Medicina, Universidad de Chile, Santiago, Chile; 8 Hospital Dr. Hernan Henríquez Aravena, Temuco, Chile; 9 Department of Pathology, Faculty of Medicine, Universidad de la Frontera, Temuco, Chile

**Keywords:** Cancer, Immunology, Gastroenterology

## Abstract

Chile has high incidence rates of gallbladder cancer globally, particularly among Amerindian women, who also have a high prevalence of gallstones. We examined differences in inflammatory biomarkers between Mapuche and non-Mapuche women from the Chile Biliary Longitudinal Study, a cohort of women with ultrasound-detected gallstones. We randomly selected 200 Mapuche women frequency matched to non-Mapuche women on age and statin use Inflammatory biomarkers were analyzed using a multiplex assay and linear regression to assess associations of a priori markers (CCL20, CXCL10, IL-6, and IL-8) with ethnicity. Novel biomarkers were analyzed using exploratory factor analysis (EFA) and sufficient dimension reduction (SDR) to identify correlated marker groups, followed by linear regression to examine their association with ethnicity. The mean values of IL-8 were higher in Mapuche than non-Mapuche women (*P* = 0.04), while CCL20, CXCL10, and IL-6 did not differ significantly by ethnicity. EFA revealed two marker groups associated with ethnicity (*P* = 0.03 and *P* < 0.001). SDR analysis confirmed correlation between the biomarkers and ethnicity. We found higher IL-8 levels among Mapuche than non-Mapuche women. Novel inflammatory biomarkers were correlated with ethnicity and should be studied further for their role in gallbladder disease. These findings may elucidate underlying ethnic disparities in gallstones and carcinogenesis among Amerindians.

## Introduction

Gallbladder cancer (GBC) is a rare, but highly fatal cancer often diagnosed in late stages with an overall median survival rate of less than 1 year^1,2^. In the U.S. and worldwide, gallbladder cancer occurs in < 2 per 100,000 individuals^3,4^. Chile has some of the highest rates in the world, and the incidence in American Indians is much higher, particularly among women. For example, the age-standardized incidence rate for Chilean women is 17.2 per 100,000 and in Mapuche Indian women the incidence is 25.0 per 100,000^5^. Mapuche ancestry is associated with a higher risk of death from gallbladder cancer as well^6^. The reason for the higher GBC incidence and mortality in Mapuche women is unclear.

Gallstones, the major risk factor for GBC, are more prevalent among women and individuals of Amerindian ancestry. The prevalence of gallstones among women of European ancestry is estimated to be 10–20%, contrasted with 49% in Mapuche women^3^. Women with Amerindian ancestry are more likely to develop gallstones earlier in life and more likely to have multiple gallstones (instead of single stones) than other ethnicities, resulting in prolonged chronic inflammation^7^. This chronic inflammatory state causes the release of inflammatory mediators, such as cytokines, chemokines, and prostaglandins, into the microenvironment. The sustained release of cytokines can lead to can lead to a pro-carcinogenic microenvironment, e.g., through promoting cellular proliferation and inhibiting apoptosis^8,9^.

Increased inflammation in response to gallstones among Native Americans may be the as yet unidentified piece of the pathogenesis process^7^. Evaluating inflammatory profiles in Amerindians may help elucidate the mechanisms involved in the pathway from gallstones to gallbladder carcinogenesis. Previous research has identified four inflammation protein markers, C–C motif chemokine ligand 20 (CCL20), C-X-C motif chemokine ligand 10 (CXCL10), interleukin (IL) 6, and IL-8, positively associated with the development of and mortality due to gallbladder cancer^10,11^. However, these inflammatory biomarkers were measured in participants with cancer, so reverse causation is a concern in these previous studies^10,11^. Evidence of inflammation related to ancestry may help inform future studies of risk stratification in high risk populations. Therefore, we examined differences in inflammatory response between presumed cancer-free women with gallbladder disease of Mapuche and non-Mapuche ancestry in Chile.

## Methods

### Study description

The Chile Biliary Longitudinal Study (BiLS) is a prospective cohort study of women aged 50–74 with ultrasound-confirmed gallstones from the southern-central region of Chile^12^. The baseline visit consisted of a detailed hepatobiliary ultrasound, physical exam, blood collection, and an interviewer-administered questionnaire that included socio-demographics, medical history, and medication use. The study was approved by institutional review boards of the United States National Cancer Institute, Pontificia Universidad Católica, and the Chilean Ministry of Health. All participants provided written consent and all methods were performed in accordance with the relevant guidelines and regulations.

From this cohort we randomly selected 200 women with self-reported Mapuche ethnicity and 200 women with self-reported Latina/Chilean (majority admixed Hispanic/European) ethnicity (non-Mapuche). To increase the specificity of ethnicity, women were required to have a paternal and maternal Mapuche surname and to self-identify as Mapuche to be included in the Mapuche group. Women were included in the non-Mapuche group if they did not have a Mapuche surname or self-identify as Mapuche. Mapuche ancestry was assessed using NextSeq 550 (Illumina Inc., San Diego, CA) to test 150 ancestry informative markers (AIM) on 380 of the women^13^. The degree of correlation between AIM and our method of self-report plus surname was quantified using the Spearman correlation coefficient^14^.

Because the use of statins is strongly associated with reduced inflammation^15^, gallstones^16^, and gallbladder cancer^17^, we stratified our sample on statin use (e.g. 100 statin users and 100 non-users from both ethnic groups). We further matched the two ethnic groups on age at enrollment in 5-year groups (50–54, 55–59, 60–64, 65–69, or 70–74). We categorized women as obese (≥ 30 kg/m^2^) or not obese (< 30 kg/m^2^) based on baseline measures of body mass index (BMI). Waist and hip circumference were measured twice by the study technicians, and the average of the two measurements was used in this analysis. Diabetes mellitus was identified by self-report of a doctor’s diagnosis. Participants were asked if they had ever experienced pain associated with biliary colic in the previous 5 years. Women’s smoking history was based on self-report and categorized as current, former, or never smoking. Education level was based on highest number of years of schooling attained (0–8, 9–12, or ≥ 13 years), and monthly family income was dichotomized as ≤ $250,000 Chilean Pesos (CLP) or > CLP$250,000 (~ $293 in US dollars). Women reported their level of coverage in Chile’s public health insurance system Fondo Nacional de Salud (FONASA), which covers approximately 80% of the Chilean population in four hierarchical levels (Groups A–D) determined by taxable income. Women also recalled their frequency of green chili, red chili, and fried food consumption in the previous 12 months.

### Laboratory methods

Serum samples (1 μL) collected at study baseline were analyzed for 92 biomarkers (Supplemental Table 1) on the Proseek Multiplex Inflammation I multiplex proximity extension assay panel (Olink Bioscience, Uppsala, Sweden) using a Fluidigm Biomark reader (Fluidigm Corporation, USA), as previously described^18^. Relative protein levels were calculated from cycle threshold values with corrections for assay variation and presented as normalized protein eXpression (NPX) on a logarithmic scale. Samples were randomly placed across testing plates. Twenty blinded duplicates were included to assess reproducibility within and between plates.

Marker values were natural log transformed. All samples had batch coefficient of variation ≤ 5% and intraclass correlation > 75%. The 4 a priori markers of interest (CCL20, CXCL10, IL-6, and IL-8) had intraclass correlations > 95%. From the 88 novel inflammation markers, we excluded the following 10 from the exploratory analyses because ≥ 90% of values were below the lower limit of detection (LLOD): interferon gamma, IL1a, IL2, IL4, IL13, IL20, IL33, leukemia inhibitory factor, thymic stromal lymphopoietin, and tumor necrosis factor. For six markers [fibroblast growth factor (FGF)-23, IL5, IL10RA, IL17A, IL17C, and IL20RA], 25–50% of values were below LLOD. Thus, these markers were categorized into tertiles. Five markers (FGF5, IL2RB, IL22RA1, and IL24) with 75–90% of values below LLOD were dichotomized as detectable or undetectable. The remaining markers were normally distributed (the smallest value the LLOD) and analyzed continuously. Further, 24 women (13 Mapuche and 11 non-Mapuche) with extreme values (> 2 standard deviations) on 2 or more markers were excluded. A total of 376 women were included in the exploratory factor analysis (EFA) and sufficient dimension reduction (SDR) described below.

### Statistical analyses

Multiple linear regression was used for the primary analysis to estimate the associations between ethnicity and the four a priori inflammatory biomarkers (CCL20, CXCL10, IL-6, and IL-8) as dependent variables. Models were adjusted for the matching variables, (age group and statin use) along with the covariates associated with ancestry and inflammation (diabetes, waist circumference, smoking status, diet, educational attainment, income, and health insurance status). Analyses were also stratified by obesity and diabetes. To assess heterogeneity between strata, we included an interaction term for ethnicity with, statin use, diabetes, or obesity separately in the models and assessed its significance using a Wald Chi-square test. In tables we present the adjusted mean differences between Mapuche and non-Mapuche women as slopes (β) and standard errors (SEs) from the regression models. A *P* value < 0.05 was considered statistically significant. Because the 4 a priori markers were tested based on strong prior hypotheses, we did not adjust for multiple comparisons^19^.

We used EFA to identify correlations between the 78 biomarkers. Inflammatory factors were extracted using the principal factor method to estimate the factor loadings (correlations) between each inflammation marker biomarker and underlying inflammatory processes based on the non-Mapuche women. We retained five inflammatory factors based on the scree plot analysis. The five inflammatory factors were rotated using a varimax rotation, which makes factors statistically independent. Factor scores (linear combinations of the markers weighted by their factor loadings) were estimated for the 376 women based on the factor loadings of the non-Mapuche women. Multiple linear regression was used to estimate the association between ethnicity and each inflammatory factor score as the dependent variable, adjusted for age, statin use, diabetes, waist circumference, smoking status, educational attainment, health coverage, and diet.

While the EFA assesses patterns between markers based on their correlations, these factors are not selected based on any marker relationships to outcome. We therefore also applied a recently developed SDR method that identifies linear combinations of markers that are most associated with the outcome (being Mapuche) and also accommodates limits of detections in the computations^20^. Due to the somewhat limited sample size, we excluded the categorical markers and restricted this analysis to the 61 continuous markers (Supplemental Table 2). We estimated two linear marker combinations that can be used to model outcome or for prediction. We plotted the two linear combinations (cLAD-1 and cLAD-2) for each person to identify clusters associated with ethnicity.

Regression analyses and EFA were conducted in SAS version 9.4 (SAS Institute, Cary, NC, USA). The SDR analysis was conducted in Matlab (MathWorks, Inc., Natick, MA, USA).

## Results

Baseline samples were selected from 400 women participating in Chile BiLS (200 Mapuche and 200 non-Mapuche women). There was strong correlation between ancestry defined by self-reported Mapuche ethnicity plus surname with genomic ancestry inferred by AIM (r = 0.81, *p* < 0.0001; Supplemental Figure). As shown in Table 1, due to sample selection and matching, the mean age was 60 years (standard deviation [SD] = 6) and statin use was equivalent (50%) in both groups. The BMI of Mapuche women (32.8 kg/m^2^ [SD = 5]) was higher than the BMI of non-Mapuche women (31.4 kg/m^2^ [SD = 6]), and a larger proportion of Mapuche women were obese (71% vs. 54%). Mapuche women had higher mean waist circumference and hip circumference than non-Mapuche women (*P* < 0.0001 and *P* = 0.02, respectively). A diabetes diagnosis was more common in non-Mapuche women (34%) than Mapuche women (27%). Non-Mapuche women were more likely to be ever smokers (45% vs. 17%) than Mapuche women. Neither the presence of multiple gallstones (45% in both groups), nor abdominal pain consistent with biliary colic (30% in Mapuche and 32% in non-Mapuche women) differed between the ancestral groups. Mapuche women were more likely to have lower educational attainment (74% vs. 48% with 0–8 years of schooling) and be covered by FONASA Group A (58% vs. 41%).

**Table 1:** Demographic and medical characteristics of Mapuche and non-Mapuche Chilean women sampled from the Chile BiLS cohort (N = 400).

Characteristic	Mapuche (N = 200)	Non-Mapuche (N = 200)	*P* value
Age in years, mean (SD)	59.5 (6)	59.7 (6)	0.66
Statin use, n (%)	99 (50)	100 (50)	0.92
Education level¸ n (%)			
0–8 years	149 (74)	96 (48)	< 0.001
9–12 years	42 (21)	88 (44)	
≥ 13 years	8 (4)	16 (8)	
Missing	0 (0)	1 (0)	
Monthly family income, n (%)			
≤ CLP$250,000	116 (58)	105 (53)	0.03
> CLP$250,000	49 (25)	72 (36)	
Missing	35 (18)	23 (12)	
Health coverage,^a^ n (%)			
FONASA Group A	115 (58)	81 (41)	< 0.001
FONASA Group B	42 (21)	82 (42)	
FONASA Group C	3 (2)	10 (5)	
FONASA Group D	9 (41)	13 (7)	
Unknown/none	29 (15)	10 (5)	
BMI in kg/m^2^, mean (SD)	32.8 (5)	31.4 (6)	0.01
Obese (BMI ≥ 30 kg/m^2^), n (%)	142 (71)	107 (54)	< 0.001
Waist circumference in cm, mean (SD)	103.2 (12)	98.2 (13)	< 0.001
Hip circumference in cm, mean (SD)	111.0 (11)	108.5 (12)	0.02
Diabetes diagnosis, n (%)	54 (27)	67 (34)	0.16
Ever smoker, n (%)	34 (17)	90 (45)	< 0.001
Gallstones, n (%)			
1 stone	71 (36)	82 (41)	0.32
≥ 2 stones	89 (45)	89 (45)	
Unknown	39 (19)	29 (14)	
Abdominal pain in past 5 years, n (%)	58 (30)	62 (32)	0.66
Diet, n (%)			
Consumption of fresh green chilis	111 (58)	87 (42)	0.002
Consumption of fresh red chilis	78 (63)	120 (44)	< 0.001
Consumption of dried red chilis	66 (55)	132 (47)	0.137
Consumption of red chili paste	61 (60)	137 (48)	0.136
Consumption of fried food	119 (53)	79 (45)	0.104
Regular use of Aspirin/NSAID	50 (25)	59 (30)	0.327

Values do not add up to the total due to missingness for the following number of participants: statin use—1; education level—1; monthly family income—56; health insurance coverage—4; smoking status—1; gallstones—1; abdominal pain—14; diet—3.

*BMI* body mass index, *CLP* Chilean pesos, *cm* centimeters, *FONASA* Fondo Nacional de Salud (Chile’s public health insurance system), *kg* kilograms, *m* meters, *n* number, *SD* standard deviation.

^a^FONASA Groups A–D are hierarchical classifications used to determine the proportion of healthcare costs covered by the government, based on taxable individual income. FONASA Group A has the lowest income level and receives the highest level of governmental coverage of healthcare costs.

### A priori markers

The results of the multivariable regression for the four inflammation markers identified a priori are shown in Table 2. Adjusting for age, statin use, diabetes, waist circumference, smoking, education, health coverage, and diet, the mean values for IL-8 were significantly higher in Mapuche women compared to non-Mapuche women (*P* = 0.04). The levels of CCL20, CXCL10, and IL-6 did not differ between the ethnic groups (*P* = 0.25, *P* = 0.07, and *P* = 0.77, respectively). The relationship between ancestry and any of the four inflammation markers was not modified by statin use, diabetes, or obesity (*P* > 0.05 for all comparisons).

**Table 2:** Multivariable associations between ethnic group and 4 a priori inflammation markers.

Marker	Mapuche mean^a^	Non-Mapuche mean^a^	β (SE)	*P* value
CCL20	7.63	7.52	0.11 (0.10)	0.275
CXCL10	10.68	10.54	0.14 (0.08)	0.068
IL-6	4.96	4.99	− 0.02 (0.08)	0.767
IL-8	7.29	7.14	0.15 (0.08)	0.044

Adjusted for age group (50–54, 55–59, 60–64, 65–69, or 70–74 years), statin use, waist circumference (in cm), diabetes, smoking status (ever or never), education (0–8, 9–12, or ≥ 13 years of schooling attained), FONASA health coverage, and diet (fresh chili and fried food consumption).

^a^Values are natural log transformed.

### Novel markers

Five inflammatory factors were identified from the EFA and account for 32% of the total variance among the 78 markers analyzed. The contribution of each inflammation marker is shown in Supplemental Table 3. The shading indicates that the marker contributed significantly to the inflammatory factor and there was very little overlap in significant markers between the inflammatory factors. Twenty markers in inflammatory factors 3 and 5 had a coefficient >|0.10|, suggesting a stronger contribution to that factor. These markers are: eukaryotic translation initiation factor 4E-binding protein 1 (4EBP1), AXIN1, Caspase-8 (CASP-8), CCL23, CCL28, delta and notch-like epidermal growth factor-related receptor (DNER0, protein S100-A12 (EN-RAGE), FGF-19, FGF-21, IL-4, IL-20RA, leukemia inhibitory factor receptor (LIF-R), matrix metalloproteinase-10 (MMP-10), oncostatin-M (OSM), SIR2-like protein 2 (SIRT-2), sulfotransferase 1A1, (ST1A1), STAM-binding protein (STAMBP), transforming growth factor alpha (TGF-α), TNF-related apoptosis-inducing ligand (TRAIL), and TNF-related activation-induced cytokine (TRANCE). After regressing ethnicity on each inflammation group, adjusting for age, statin use, waist circumference, diabetes, smoking status, education, FONASA health coverage, and diet, inflammatory factor 3 was positively correlated with Mapuche ethnicity (*P* = 0.03) and inflammatory factor 5 was negatively correlated with Mapuche ethnicity (*P* < 0.001) (Table 3).

**Table 3:** Association between Mapuche ethnicity and inflammatory factors from exploratory factor analysis.

Variable	β (SE)	*P* value
Inflammatory factor 1	− 0.09 (0.10)	0.377
Inflammatory factor 2	0.13 (0.11)	0.224
Inflammatory factor 3	0.23 (0.11)	0.032
Inflammatory factor 4	− 0.10 (0.09)	0.263
Inflammatory factor 5	− 0.34 (0.11)	< 0.001

Multiple linear regression model adjusted for age group (50–54, 55–59, 60–64, 65–69, or 70–74 years), statin use, waist circumference (in cm), diabetes, smoking status (ever or never), education (0–8, 9–12, or ≥ 13 years of schooling attained), FONASA health coverage, and diet (fresh chili and fried food consumption).

**Figure 1:**
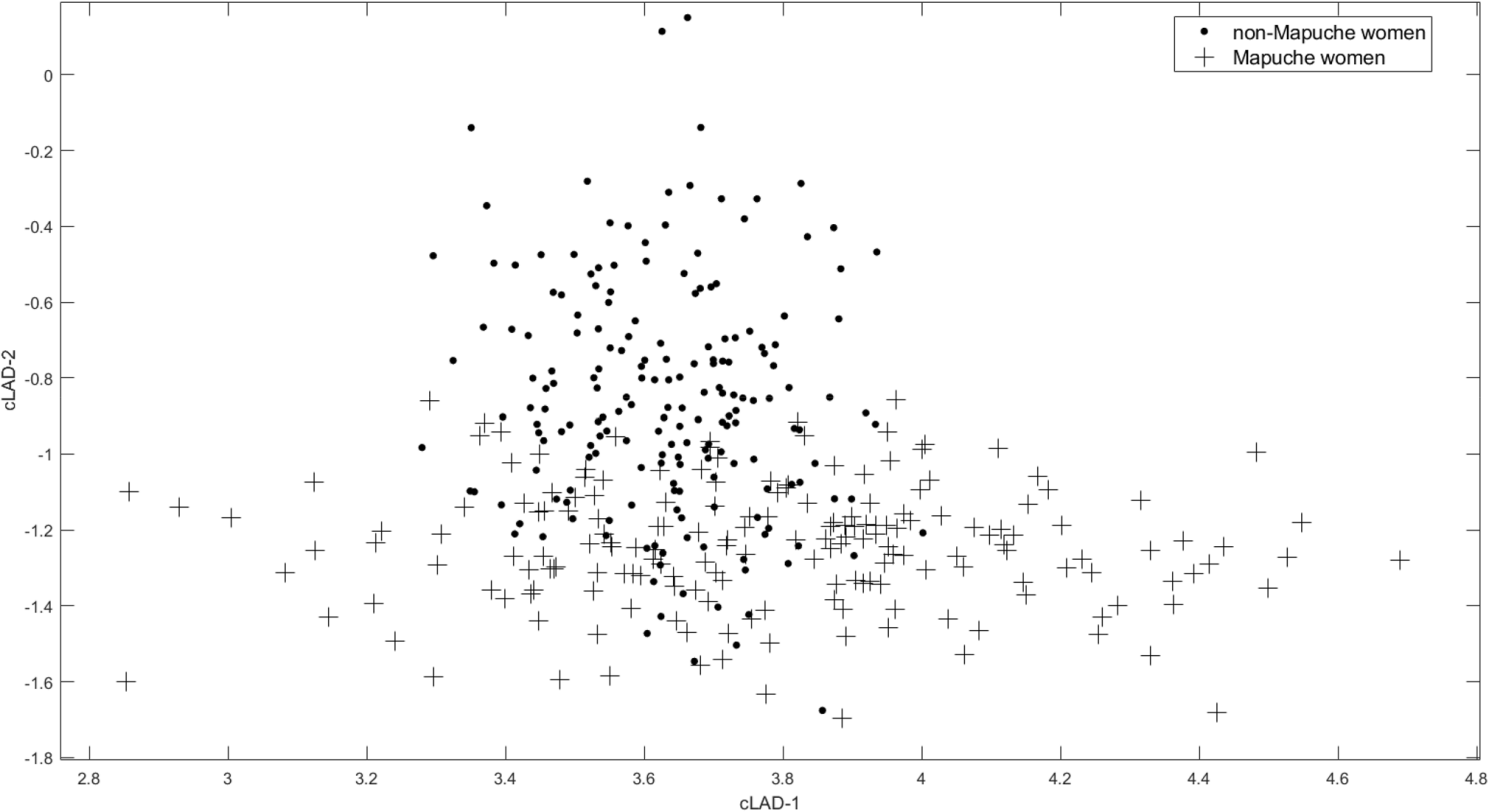
Projections in coordinate system defined by the two cLAD linear marker combinations Crosses represent Mapuche women and dots represent non-Mapuche women.

Figure 1 shows the women in the study plotted in the coordinate system given by the two linear SDR combinations of the 61 markers. It can be seen that the Mapuche women separate well visually from the non-Mapuche. No markers were eliminated from the linear combinations when we incorporated variable selection into the model, suggesting all markers were important in the estimation.

## Discussion

We conducted a comprehensive analysis of differences in circulating levels of inflammatory proteins between women of Mapuche and Chilean ancestry with gallbladder disease in Chile using multiplexed assays. IL-8 levels were differentially expressed by ethnicity among women with gallbladder disease, and this difference was not modified by diabetes or obesity. Our finding suggests that IL-8 may be an important biomarker in the gallbladder disease process. Using the 78 novel biomarkers, we found two inflammatory processes were associated with ethnicity. Further, the SDR analysis suggests that these inflammatory markers are good at differentiating between Mapuche and non-Mapuche women. These markers may reflect an underlying biologic process that explains some of the increased prevalence in gallstones and gallbladder cancer seen between Mapuche and non-Mapuche. Additional work is needed to understand the inflammatory processes involved in the pathway between gallstones and gallbladder carcinogenesis.

IL-8 was significantly upregulated among Mapuche women compared to non-Mapuche women in our study. A chemoattractant that recruits lymphocytes, dendritic cells, neutrophils, and monocytes to sites of infection, IL-8 attracts neutrophils through chemokine receptors CXCR1 and CXCR2, which induces hepatocyte necrosis through the release of reactive oxygen species and proteases^21^. IL-8 is notable as a potential biomarker of tumor aggressiveness, as increased serum levels are associated with poor prognosis in both liver and gallbladder cancer^11,21,22^.

Chemokine pathways involving CXCL10 in the context of the tumor microenvironment of hepatocellular carcinoma have been found to recruit immune cells with anti-tumor activity^23^. However, differential expression of the same biomarker in the serum or within the tumor could reflect different biological processes^24^. IL-6 may be an important mediator between the chronic inflammatory state due to gallstones and progression to carcinogenesis^25^. It has previously been shown to be elevated in gallbladder cancer patients relative to those with gallstones^10^ as its overexpression is associated with poor tumor differentiation, local invasion, metastasis, and survival^11,25^. In the present study, while IL-6 levels were elevated above clinically normal levels^26^ in both groups likely due to gallstone disease, its expression did not differ between ethnic groups, suggesting this biomarker is not strongly related to ancestry.

TRAIL has been previously identified as important markers in gallbladder cancer survival^11,27^, TRAIL and TNF-α are cytokines that bind to death receptors and recruit CASP-8 which triggers cell death inducing apoptosis in cancer cells^28^. Lower levels of circulating TRAIL were found in patients with GBC compared to those with gallstones^27^, and higher levels TRAIL were associated with increased survival after GBC diagnosis^11^. These findings could indicate that TRAIL expression may be immunoprotective. Further, both TRAIL and TNF-α are thought to be importer mediators of inflammation of adipose tissue and obesity-related disease^29^. Thus, while these biomarkers could also represent inflammatory processes related to metabolic diseases, these differences persisted with adjustment for diabetes and waist circumference^30^. Lower levels of *TRAF3* gene, a member of the TNF receptor-associated factor (TRAF) protein family, have been found in individuals with gallstones^31,32^. These findings suggest that *TRAF3* expression contributes to the inflammatory response in the development of gallstone disease. Future research may focus on ancestral differences in *TRAF3* risk allele frequency^32^.

Several novel biomarkers used in the EFA have important roles in the inflammatory process and carcinogenesis. For instance, increased levels of FGF-19 the sera of patients with extrahepatic cholestasis suggests that cholestasis may stimulate FGF-19 production and provides evidence that this marker is involved in tumor development in the liver^33^. AXIN1 is an important component of the Wnt signal transduction pathway, a molecular pathway implicated in liver carcinogenesis. Here, AXIN1 promotes β-catenin degradation, suggesting that it functions as a tumor suppressor^34^. EN-RAGE is a proinflammatory cytokine that binds to its receptor RAGE which is expressed predominately on immune cells, endothelial and vascular smooth muscle cells, and cancer cells^35^. This binding activates an inflammatory cascade through the NF-κB signaling pathway, which is involved in several proinflammatory conditions such as type 2 diabetes^36^ and coronary heart disease^37^, and contributes a pro-tumorigenic microenvironment^35^. Other inflammatory biomarkers we identified from the EFA have been shown to promote cancer progression (CCL23^38^, CCL28^39^, DNER^40^, FGF21^41^, IL-4^42^, LIFR^43^, MMP-10^44^, OSM^45^, ST1A1^46,47^, and TRANCE^48^) or have been implicated in inhibition of tumorigenesis (AXIN1^34^, SIRT-2^49^, and 4E-BP1^50^).

A strength of our study is the use of self-reported ethnicity plus surname to identify Mapuche and non-Mapuche women. This method was highly correlated to the AIM, suggesting that it adequately captures genetic ethnicity. We also matched the women on important variables related to inflammation such as age and statin use. However, we were not able to adjust for important social and cultural differences between ethnicity groups that may also be related to inflammation, such as the use of traditional medicine^51^. This study was a cross-sectional analysis of cohort of Chilean women with gallstones. As such, we do not yet know who will develop cancer, and so cannot link the inflammatory profile to cancer outcome.

The findings of this study are an important first step to address the racial disparities in gallbladder disease and cancer among Amerindians. Native American communities have higher rates of gallstones, gallstone-related cancers, and mortality overall^8^. We found evidence that IL8 is upregulated among Mapuche women and that several novel biomarkers may be important in explaining ancestral differences among women with gallbladder disease. Additional work is needed to clarify the biologic processes involved; these findings provide insight into the pathologic process of gallstone disease in high-risk populations. Therefore, understanding the pathogenic process of gallstone disease in these populations is key to identifying targets for prevention.

## Supplementary Information

Supplementary Information
