# Supplementary material for: Inflammatory profiles in Chilean Mapuche and non-Mapuche women with gallstones at risk of developing gallbladder cancer
Source: Sci Rep. 2021 Feb 11;11:3686. doi: 10.1038/s41598-021-83300-2 (PMC7878792; doi:10.1038/s41598-021-83300-2)
Supplement: Supplementary file 1 — Supplementary Information [file 41598_2021_83300_MOESM1_ESM.docx]

**Inflammatory profiles in Chilean Mapuche and non-Mapuche women with gallstones at risk of developing gallbladder cancer**

Sarah S. Jackson,^1*^ Vanessa Van De Wyngard,^2,3^ Ruth M. Pfeiffer,^1^ Paz Cook, ^2,3^ Allan Hildesheim,^1^, Ligia A. Pinto,^4^ Sharon H. Jackson,^5^ Kelvin Choi,^5^ Ricardo A. Verdugo,^6,7^ Mara Cuevas,^6^ Cristian Yáñez,^6^ Eduardo Tobar-Calfucoy,^6^ Rocío Retamales-Ortega,^6^ Juan Carlos Araya,^3,8,9^ Catterina Ferreccio,^2,3^ and Jill Koshiol^1^

^1^Division of Cancer Epidemiology and Genetics, National Cancer Institute, Rockville, MD, USA

^2^School of Medicine, Pontificia Universidad Catolica de Chile, Santiago, Chile

^3^Advanced Center for Chronic Diseases (ACCDiS), FONDAP, Santiago, Chile
^4^Frederick National Laboratory for Cancer Research, National Cancer Institute, Frederick, MD, USA
^5^Division of Intramural Research, National Institute on Minority Health and Health Disparities, Bethesda, MD, USA
^6^Programa de Genética Human, ICBM, Facultad de Medicina, Universidad de Chile, Santiago, Chile
^7^Departamento de Oncología Básico Clínica, Facultad de Medicina, Universidad de Chile, Santiago, Chile ^8^Hospital Dr. Hernan Henríquez Aravena, Temuco, Chile
^9^ Department of Pathology, Faculty of Medicine, Universidad de la Frontera, Temuco, Chile

***Corresponding author**:
Sarah S. Jackson,
Infections and Immunoepidemiology Branch
Division of Cancer Epidemiology and Genetics
National Institutes of Health, National Cancer Institute
9609 Medical Center Dr, Rm 6-E210
Rockville, Maryland 20850, USA
E-mail: [sarah.jackson@nih.gov](mailto:sarah.jackson@nih.gov)

**Running title**: Inflammatory Profile of Amerindians with Gallbladder disease

**Key Words**: Ancestry, gallstones, Chile, Mapuche, gallbladder disease, inflammation

**Word count**: abstract – 200; text – 2,870

**Tables**: 3; 1 Figure

**Supplemental file**: 3 Tables ; 1 Figure

**Supplemental Figure**. The proportion of genetic ancestry from reference samples (left) and 380 selected samples from Chile Biliary Longitudinal Study (BiLS) (right). Each shade represents a component of predicted ancestry: red for African, green for European, light blue for Aymara (northern Amerindians), and orange for Mapuche (southern-central Amerindians).


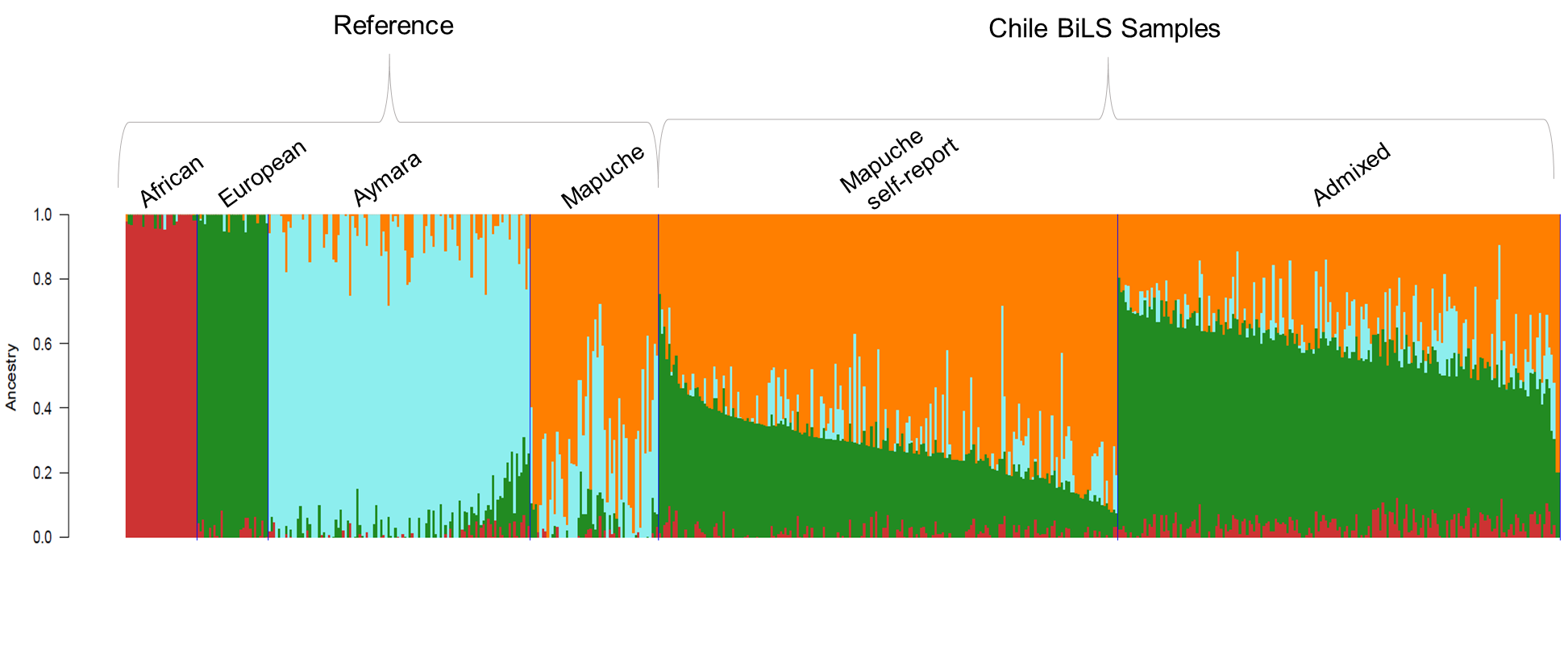


| **Supplementary Table 1.** List of inflammation markers on the Olink panel (N=92) | | | |
| --- | --- | --- | --- |
| **Protein** | **ID** | **Protein** | **ID** |
| Artemin (ARTN) | Q5T4W7 | Osteoprotegerin (OPG) | O00300 |
| Axin-1 (AXIN1) | O15169 | Urokinase-type plasminogen activator (uPA) | P00749 |
| C-C motif chemokine 25 (CCL25) | O15444 | Monocyte chemotactic protein 1 (MCP-1) | P13500 |
| C-C motif chemokine 28 (CCL28) | Q9NRJ3 | Adenosine Deaminase (ADA) | P00813 |
| CUB domain-containing protein 1 (CDCP1) | Q9H5V8 | Caspase-8 (CASP-8) | Q14790 |
| C-X-C motif chemokine 6 (CXCL6) | P80162 | C-C motif chemokine 4 (CCL4) | P13236 |
| Cystatin D (CST5) | P28325 | C-C motif chemokine 19 (CCL19) | Q99731 |
| Delta and Notch-like epidermal growth factor-related receptor (DNER) | Q8NFT8 | C-C motif chemokine 20 (CCL20) | P78556 |
| Eukaryotic translation initiation factor 4E-binding protein 1 (4E-BP1) | Q13541 | C-C motif chemokine 23 (CCL23) | P55773 |
| Fibroblast growth factor 5 (FGF-5) | Q8NF90 | CD40L receptor (CD40) | P25942 |
| Fibroblast growth factor 19 (FGF-19) | O95750 | C-X-C motif chemokine 5 (CXCL5) | P42830 |
| Fms-related tyrosine kinase 3 ligand (Flt3L) | P49771 | C-X-C motif chemokine 9 (CXCL9) | Q07325 |
| Interleukin-2 receptor subunit beta (IL-2RB) | P14784 | C-X-C motif chemokine 10 (CXCL10) | P02778 |
| Interleukin-10 receptor subunit alpha (IL-10RA) | Q13651 | C-X-C motif chemokine 11 (CXCL11) | O14625 |
| Interleukin-10 receptor subunit beta (IL-10RB) | Q08334 | Fractalkine (CX3CL1) | P78423 |
| Interleukin-12 subunit beta (IL-12B) | P29460 | Interferon gamma (IFN-gamma) | P01579 |
| Interleukin-15 receptor subunit alpha (IL-15RA) | Q13261 | Interleukin-1 alpha (IL-1 alpha) | P01583 |
| Interleukin-17A (IL-17A) | Q16552 | Interleukin-2 (IL-2) | P60568 |
| Interleukin-17C (IL-17C) | Q9P0M4 | Interleukin-4 (IL-4) | P05112 |
| Interleukin-18 receptor 1 (IL-18R1) | Q13478 | Interleukin-7 (IL-7) | P13232 |
| Interleukin-20 (IL-20) | Q9NYY1 | Interleukin-8 (IL-8) | P10145 |
| Interleukin-20 receptor subunit alpha (IL-20RA) | Q9UHF4 | Interleukin-13 (IL-13) | P35225 |
| Interleukin-22 receptor subunit alpha-1 (IL-22 RA1) | Q8N6P7 | Interleukin-33 (IL-33) | O95760 |
| Interleukin-24 (IL-24) | Q13007 | Latency-associated peptide transforming growth factor beta-1 (LAP TGF-beta-1) | P01137 |
| Leukemia inhibitory factor (LIF) | P15018 | Macrophage colony-stimulating factor 1 (CSF-1) | P09603 |
| Leukemia inhibitory factor receptor (LIF-R) | P42702 | Monocyte chemotactic protein 2 (MCP-2) | P80075 |
| Matrix metalloproteinase-1 (MMP-1) | P03956 | Monocyte chemotactic protein 3 (MCP-3) | P80098 |
| Matrix metalloproteinase-10 (MMP-10) | P09238 | Monocyte chemotactic protein 4 (MCP-4) | Q99616 |
| Neurotrophin-3 (NT-3) | P20783 | Natural killer cell receptor 2B4 (CD244) | Q9BZW8 |
| Neurturin (NRTN) | Q99748 | Programmed cell death 1 ligand 1 (PD-L1) | Q9NZQ7 |
| Oncostatin-M (OSM) | P13725 | T-cell surface glycoprotein CD5 (CD5) | P06127 |
| Protein S100-A12 (EN-RAGE) | P80511 | Tumor necrosis factor (Ligand) superfamily, member 12 (TWEAK) | O43508 |
| Signaling lymphocytic activation molecule (SLAMF1) | Q13291 | Tumor necrosis factor (TNF) | P01375 |
| SIR2-like protein 2 (SIRT2) | Q8IXJ6 | Tumor necrosis factor ligand superfamily member 14 (TNFSF14) | O43557 |
| STAM-binding protein (STAMPB) | O95630 | Tumor necrosis factor receptor superfamily member 9 (TNFRSF9) | Q07011 |
| Sulfotransferase 1A1 (ST1A1) | P50225 | Beta-nerve growth factor (Beta-NGF) | P01138 |
| T cell surface glycoprotein CD6 isoform (CD6) | Q8WWJ7 | Brain-derived neurotrophic factor (BDNF) | P23560 |
| Thymic stromal lymphopoietin (TSLP) | Q969D9 | Glial cell line-derived neurotrophic factor (GDNF) | P39905 |
| TNF-beta (TNFB) | P01374 | Transforming growth factor alpha (TGF-alpha) | P01135 |
| TNF-related activation-induced cytokine (TRANCE) | O14788 | Hepatocyte growth factor (HGF) | P14210 |
| Fibroblast growth factor 23 (FGF-23) | Q9GZV9 | TNF-related apoptosis-inducing ligand (TRAIL) | P50591 |
| Fibroblast growth factor 21 (FGF-21) | Q9NSA1 | Vascular endothelial growth factor A (VEGF-A) | P15692 |
| C-C motif chemokine 3 (CCL3) | P10147 | Interleukin-10 (IL-10) | P22301 |
| C-X-C motif chemokine 1 (CXCL1) | P09341 | Interleukin-5 (IL-5) | P05113 |
| Interleukin-18 (IL-18) | Q14116 | Eotaxin (CCL11) | P51671 |
| Stem cell factor (SCF) | P21583 | Interleukin-6 (IL-6) | P05231 |

| **Supplemental Table 2**. List of inflammatory markers included in the sufficient dimension reduction analysis | | | |
| --- | --- | --- | --- |
| ADA | CDCP1 | IL10 | SCF |
| AXIN1 | CSF1 | IL18 | SIRT2 |
| Β-NGF | CST5 | IL7 | SLAMF1 |
| CASP8 | CX3CL1 | IL12B | ST1A1 |
| CCL11 | CXCL1 | IL15RA | STAMBP |
| CCL19 | CXCL11 | IL18R1 | TGF-α |
| CCL23 | CXCL5 | LAP TGF-β-1 | TNFB |
| CCL25 | CXCL6 | LIF-R | TNFRSF9 |
| CCL28 | CXCL9 | MCP-1 | TNFSF14 |
| CCL3 | DNER | MCP-2 | TRAIL |
| CCL4 | EN-RAGE | MCP-3 | TRANCE |
| CD244 | FGF19 | MCP-4 | TWEAK |
| CD40 | FGF21 | MMP10 | UPA |
| CD5 | FLT3L | OPG | VEGFA |
| CD6 | GDNF | OSM | 4E-BP1 |
| CD8A | HGF | PD-L1 |  |

| **Supplemental Table 3.** Scoring coefficients for 78 inflammation markers in the exploratory factor analysis | | | | | |
| --- | --- | --- | --- | --- | --- |
| **Marker** | **Inflammatory**  **Factor 1** | **Inflammatory**  **Factor 2** | **Inflammatory**  **Factor 3** | **Inflammatory**  **Factor 4** | **Inflammatory**  **Factor 5** |
| TNFRSF9 | 0.10 | -0.04 | -0.01 | -0.05 | 0.07 |
| CD5 | 0.10 | -0.05 | 0.01 | -0.04 | 0.08 |
| CSF1 | 0.08 | -0.03 | -0.01 | 0.02 | 0.04 |
| IL-12B | 0.09 | -0.05 | 0.00 | -0.04 | 0.03 |
| IL-15RA | 0.07 | -0.02 | 0.01 | -0.01 | 0.03 |
| IL-10RB | 0.08 | -0.05 | -0.02 | -0.01 | 0.07 |
| CD40 | 0.05 | 0.01 | 0.00 | -0.01 | 0.05 |
| SLAMF1 | 0.07 | -0.02 | -0.02 | 0.03 | -0.05 |
| CX3CL1 | 0.08 | 0.01 | -0.04 | -0.10 | 0.01 |
| IL-18 | 0.07 | -0.05 | 0.01 | 0.09 | -0.08 |
| CD244 | 0.06 | 0.01 | 0.03 | -0.05 | -0.03 |
| PDL1 | 0.05 | -0.01 | 0.00 | 0.04 | 0.00 |
| UPA | 0.06 | 0.00 | 0.00 | -0.01 | -0.07 |
| CD6 | 0.07 | -0.03 | 0.03 | -0.03 | -0.02 |
| CCL3 | 0.04 | -0.01 | 0.01 | 0.07 | 0.04 |
| OPG | 0.04 | 0.01 | 0.00 | 0.07 | -0.11 |
| CST5 | 0.06 | 0.01 | -0.01 | -0.08 | -0.02 |
| LIFR | 0.04 | 0.03 | -0.02 | 0.05 | -0.15 |
| TRAIL | 0.05 | 0.02 | -0.03 | -0.09 | 0.11 |
| TNFB | 0.06 | 0.00 | 0.01 | -0.06 | -0.05 |
| CXCL9 | 0.05 | -0.02 | 0.03 | 0.00 | -0.02 |
| IL-10 | 0.04 | -0.02 | -0.02 | 0.05 | 0.03 |
| CD8A | 0.06 | -0.03 | -0.01 | -0.02 | 0.04 |
| CDCP1 | 0.04 | -0.02 | 0.01 | 0.12 | -0.12 |
| GDNF | 0.03 | 0.02 | -0.03 | 0.07 | -0.06 |
| β-NGF | 0.03 | -0.01 | 0.00 | 0.06 | -0.02 |
| CCL25 | 0.04 | 0.00 | -0.02 | -0.02 | -0.01 |
| FLT3L | 0.03 | 0.02 | 0.01 | -0.01 | -0.09 |
| CCL19 | 0.03 | -0.01 | 0.00 | -0.02 | 0.07 |
| IL-22RA1 | 0.02 | -0.01 | 0.00 | 0.03 | 0.00 |
| IL-24 | 0.02 | 0.00 | -0.02 | 0.01 | -0.01 |
| CXCL6 | -0.03 | 0.10 | -0.03 | 0.01 | 0.01 |
| MCP-4 | -0.03 | 0.10 | 0.00 | -0.01 | -0.01 |
| LAPTGF-β1 | -0.04 | 0.09 | 0.01 | 0.03 | 0.00 |
| TWEAK | -0.02 | 0.12 | 0.02 | -0.14 | -0.04 |
| CXCL1 | -0.05 | 0.11 | -0.02 | -0.03 | 0.02 |
| CXCL5 | -0.02 | 0.11 | -0.03 | -0.06 | -0.02 |
| CCL11 | -0.01 | 0.10 | -0.05 | -0.02 | -0.02 |
| MCP1 | 0.00 | 0.07 | -0.02 | -0.01 | 0.03 |
| CCL28 | -0.02 | 0.10 | -0.03 | 0.02 | -0.12 |
| CCL4 | -0.01 | 0.06 | 0.01 | 0.04 | 0.01 |
| IL-7 | -0.02 | 0.08 | -0.02 | -0.03 | 0.01 |
| CXCL11 | 0.00 | 0.05 | 0.02 | 0.03 | -0.04 |
| MCP-2 | 0.00 | 0.07 | 0.00 | -0.04 | -0.02 |
| DNER | -0.02 | 0.11 | 0.00 | -0.09 | -0.14 |
| TNFSF-14 | -0.07 | 0.05 | 0.06 | 0.10 | 0.06 |
| VEGFA | -0.02 | 0.05 | -0.01 | 0.02 | 0.07 |
| TGF-α | -0.05 | 0.05 | 0.00 | 0.08 | 0.13 |
| FGF-5 | 0.00 | 0.06 | -0.01 | -0.03 | -0.02 |
| MCP-3 | -0.01 | 0.03 | 0.00 | 0.08 | 0.04 |
| FGF-23 | 0.00 | 0.02 | -0.01 | 0.05 | 0.04 |
| IL-2RB | 0.00 | 0.03 | -0.01 | 0.00 | -0.01 |
| NT-3 | 0.01 | 0.03 | -0.02 | -0.04 | 0.00 |
| FGF-19 | 0.01 | 0.01 | 0.00 | -0.03 | 0.04 |
| SIRT-2 | 0.01 | -0.03 | 0.18 | -0.04 | -0.02 |
| STAMBP | 0.01 | -0.03 | 0.18 | -0.05 | -0.03 |
| 4EBP1 | 0.03 | -0.05 | 0.16 | -0.04 | -0.03 |
| CASP-8 | 0.01 | -0.03 | 0.15 | -0.01 | 0.00 |
| AXIN1 | -0.03 | -0.01 | 0.14 | -0.01 | 0.04 |
| ST1A1 | -0.03 | -0.01 | 0.12 | 0.04 | 0.02 |
| EN-RAGE | -0.04 | 0.00 | 0.10 | 0.05 | 0.02 |
| ADA | 0.03 | 0.00 | 0.09 | -0.01 | -0.09 |
| IL-17A | 0.00 | 0.05 | -0.07 | 0.00 | 0.01 |
| HGF | -0.02 | 0.02 | 0.01 | 0.15 | 0.02 |
| IL-18R1 | 0.03 | -0.03 | 0.00 | 0.14 | -0.05 |
| OSM | -0.07 | 0.05 | 0.01 | 0.12 | 0.11 |
| NRTN | 0.01 | -0.03 | -0.02 | 0.11 | 0.01 |
| IL-5 | -0.02 | -0.01 | 0.00 | 0.10 | 0.02 |
| IL10RA | -0.01 | 0.01 | 0.00 | 0.09 | -0.07 |
| ARTN | 0.00 | -0.01 | 0.02 | 0.04 | -0.02 |
| SCF | 0.01 | 0.07 | 0.01 | -0.20 | 0.04 |
| TRANCE | 0.02 | 0.00 | 0.00 | -0.07 | 0.17 |
| MMP-10 | 0.01 | 0.01 | -0.05 | 0.00 | 0.15 |
| FGF-21 | 0.00 | -0.02 | -0.02 | 0.07 | 0.13 |
| CCL-23 | 0.03 | 0.02 | 0.01 | -0.13 | 0.12 |
| IL-17C | 0.02 | 0.01 | -0.03 | -0.03 | 0.09 |
| IL-4 | 0.00 | 0.01 | -0.01 | 0.02 | -0.12 |
| IL-20RA | 0.01 | 0.03 | 0.00 | -0.01 | -0.13 |
| Note: Grey highlighting indicates markers that contributed significantly to that factor. | | | | | |
